# A novel insight into membrane fouling mechanism regarding gel layer filtration: Flory-Huggins based filtration mechanism

**DOI:** 10.1038/srep33343

**Published:** 2016-09-15

**Authors:** Qian Lei, Meijia Zhang, Liguo Shen, Renjie Li, Bao-Qiang Liao, Hongjun Lin

**Affiliations:** 1 College of Geography and Environmental Sciences, Zhejiang Normal University, Jinhua, 321004, P.R. China; 2 Department of Chemical Engineering, Lakehead University, 955 Oliver Road, Thunder Bay, Ontario P7B 5E1, Canada

## Abstract

This study linked the chemical potential change to high specific filtration resistance (SFR) of gel layer, and then proposed a novel membrane fouling mechanism regarding gel layer filtration, namely, Flory-Huggins based filtration mechanism. A mathematical model for this mechanism was theoretically deduced. Agar was used as a model polymer for gel formation. Simulation of the mathematical model for agar gel showed that volume fraction of polymer and Flory-Huggins interaction parameter were the two key factors governing the gel SFR, whereas, pH and ionic strength were not related with the gel SFR. Filtration tests of gel layer showed that the total SFR value, effects of pH and ionic strength on the gel SFR well agreed with the perditions of model’s simulation, indicating the real occurrence of this mechanism and the feasibility of the proposed model. This mechanism can satisfactorily explain the extremely high SFR of gel layer, and improve fundamental insights into membrane fouling regarding gel layer filtration.

Emerging as a highly efficient technology for wastewater treatment and reclamation, membrane bioreactor (MBR) has attracted considerable attention, and being increasingly used in various applications^1^,^2^. However, membrane fouling remains the major obstacle limiting its universal application^3^,^4^. To date, exploring membrane fouling mechanisms and the control strategies is still the primary interest for MBR^5^,^6^ as well as other membrane processes research^7^,^8^,^9^.

Long-term operation of a MBR system could generally lead to formation of a foulant layer on membrane surface, which could be subdivided into cake layer and gel layer. In some cases, gel layer was inevitable formed, and was the major contributor to membrane fouling^10^,^11^,^12^. The foulant layer formed on membrane surface, as well as membrane used in MBRs, is a kind of porous media. Being a porous media, cake layer possessed filtration resistance at least 10 times higher than that of ultrafiltration/microfiltration membrane^13^,^14^. Moreover, it was reported that specific filtration resistance (SFR) of cake layer was varied from magnitude of 10^13^ to 10^14 ^m·kg^−1 ^14,15,16, whereas, SFR of gel layer could be as high as magnitude of 10^15^ to 10^16 ^m·kg^−1 ^11,12, and gel layer directly formed by SMP or model foulants even possessed a SFR at magnitude of 10^17^ to 10^18 ^m·kg^−1 ^17,18. At same time, scanning electron microscopy (SEM) observation has confirmed that gel layer possessed high measured porosity^19^,^20^. Gel layer had significantly higher water content than cake layer^12^. According to conventional filtration theory expressed by Carman-Kozeny equation^21^, high porosity results in low filtration resistance. The big gap of SFR between cake layer and gel layer has puzzled the research community concerning membrane fouling for a long time.

Attempts have been devoted into investigating the mechanisms underlying the contradiction between filtration behavior and structure of gel layer. Apparently, conventional filtration theory cannot explain this contradiction. Wang and Waite^11^ attributed multivalent metal complexation to the cause of high SFR of gel layer. As a fact, for some model foulants used for gel layer formation, the formation process could be totally independent of multivalent metals as pure water was used, while the gel layer possessed unusually high filtration resistance simultaneously. Therefore, this cannot be considered as the universal mechanism of high SFR of gel layer. It was also suspected that lack of connectivity between pores maybe the exact cause responsible for the high SFR of gel layer^22^. However, the gel layer formed during filtration process was generally very thin^10^,^11^,^12^, which should present certain connectedness. Moreover, it was observed that gel layer possessed pores in dimension of micron size range^19^,^20^, obviously, there is no particular mechanism to block or disconnect such size pores in the thin gel layer. Thus, lack of connectivity could not be the likely mechanism. Another suspected mechanism recently proposed to explain the high SFR of gel layer is a kind of osmotic pressure effect^12^. This kind of osmotic pressure effect was suggested to stem from retaining counter-ions in the matrix of gel layer due to negatively charged functional groups carried by proteins prevailing in gel layer. The retained counter-ions cause the low chemical potential of water in gel layer as compared with permeate, inducing an osmotic pressure-induced resistance when filtration through gel layer^23^,^24^. However, a conflicted fact to this mechanism is that the main component implicit in gel formation has been identified to be the polysaccharides rather than proteins^11^, and polysaccharides can be almost electro-neutrality. Thus, this kind of osmotic pressure effect cannot satisfactorily explain the high SFR for gel layer, especially for the gel formed by polysaccharides. As all the organic macromolecules prevailing in MBRs regardless of proteins and polysaccharides have somewhat gelling propensity^20^, it is quite desirable to propose a proper mechanism for better understanding and control of membrane fouling.

Field and Aimar^25^ found that the activity of water on the boundary layer next to the gel layer was not equal to the activity of water in the bulk, and developed a model including the influence of both osmotic pressure and the variation in viscosity due to concentration polarization. This work can be viewed as a start point of this study. Based on theoretical analysis, Keiding *et al*.^26^ introduced “chemical potential” conception into sludge dewatering process study, and indeed gave significant insight into mechanisms of sludge dewatering process. Filtration process was considered to be somewhat similar to dewatering process. Moreover, it was reported that gel layer formation from polymers involved significant chemical potential variation^27^,^28^, which can be described by Flory-Huggins theory^29^,^30^. Implicated by these studies, it is reasonable to assert that analysis from chemical potential viewpoint is very conductive to propose a suitable mechanism responsible for the gel layer filtration behavior. In addition, the related critical parameters and experimental verification of the real occurrence of the proposed mechanism merit investigation.

This study, therefore, aims to link chemical potential variation to filtration resistance of gel layer, and then to propose a novel mechanism describing the filtration resistance of gel layer. In practice, mathematical models were firstly formulated, and effects of the major parameters were theoretically analyzed. Thereafter, agar was used as a model of polysaccharides for gel formation. Filtration tests of agar gel layer were performed. The model was simulated with the conditions of filtration tests. In order to verify the real occurrence of this mechanism, experimental results were compared with predictions of the model simulation. This study will enhance fundamental insights into fouling mechanisms regarding gel filtration.

## Material and Methods

### Agar gel preparation

Agar (Sigma, St Louis, MO) was selected as model polysaccharides for gel formation. Agar is the most commonly used model material for gel study. Different from proteins and some other materials, it has very low surface charge. Selecting agar as model gel material can minimize the effects of surface charge on SFR. The agar gel was prepared by addition of certain amount of agar powder into the ultrapure water, followed by heating with a microwave oven for 11 min. The agar gel solution was prepared after it was cooled below 30 °C.

### Evaluation of filtration resistance

Filtration tests of agar gel were performed in a stirred cell (MSC 300, Shanghai SINP Membrane Science Technology Co. Ltd., China) with 50 kPa constant applied pressure. The cell has effective filtration area of 0.00332 m^2^ with effective volume of 300 mL. Permeate production during filtration was recorded by an electronic balance. Darcy’s law describes the relationship among applied pressure (ΔP), filtration flux (J), dynamic viscosity of permeate (*σ*) and filtration resistance.


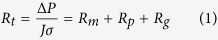


where R_t_, R_m_, R_p_ and R_g_ are total filtration resistance, membrane filtration resistance, pore clogging filtration resistance and gel layer filtration resistance, respectively. For the gel layer filtration, R_p_ can be ignored as no obvious pore clogging in membrane occurs. The experimental procedure to obtain the values of other resistance components can refer to Lin *et al*.^31^. The specific filtration resistance (SFR) of the agar gel was calculated by


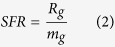


where m_g_ is the dry weight of gel layer.

In order to investigate the effects of pH and ionic strength on the SFR of gel, series water solutions with pH of 3.0, 5.0, 7.0 and 9.0, and ionic strength of 0 and 0.35 mol·L^−1^ were firstly prepared. Thereafter, 50 ml agar gel solution (0.03 g·L^−1^) which has been adjusted to the desired pH and ionic strength was subjected to filtration to form a thin gel layer in the stirred cell. The prepared water solution was then filtrated through the prepared gel layer. By recording the permeate production, the SFR of the gel layer can be calculated according to Eqs 1 and 2.

### Experiments regarding heating gel process

Experiments regarding heating gel process were conducted to verify the existence of Flory-Huggins based mechanism. Agar gel with concentration of 3 g·L^−1^ was first prepared. Four prepared agar gel samples with certain weight were placed in an oven at 35 °C, and then the weight of samples were recorded at intervals of one hour. The lost water content for each sample at different interval can be calculated.

### Thermogravimetric analysis (TGA) of gels

The agar gels were made at room temperature in a stirred filtration cell under 50 kPa. Then the prepared gels were put in an oven with 60 °C for 12 hours before thermogravimetric analysis (TGA). TGA runs were carried out in a temperature range of 0–600 °C using a TA SDT Q600 TGA (TA Instruments, USA), under nitrogen atmosphere, at a constant heating rate of 10 °C/min. The residual weight of the gels was recorded.

### Statistical analysis

Statistical analysis was conducted using SPSS software V18.0. Significance of the difference between treatment means was checked by an analysis of variance (ANOVA) method. The difference between two means was considered to be significant when P < 0.05.

## Results and Discussion

### Theoretical analysis and modeling of filtration through gel layer

**Figure 1:**
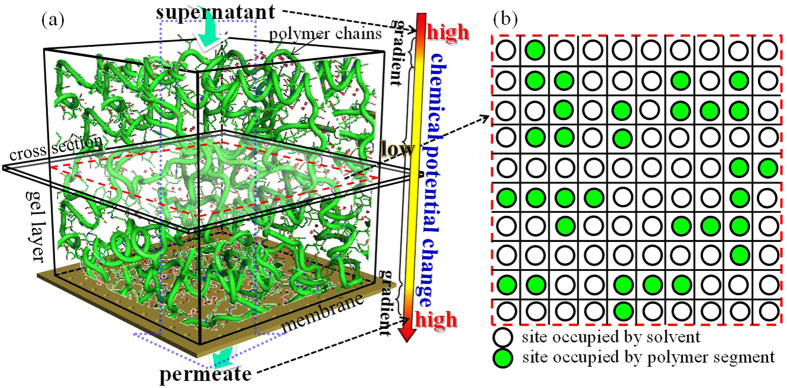
Schematic of (**a**) 3D structure of gel layer, (**b**) lattice of cross section of gel layer.

Figure 1a shows the schematic of 3D structure of a gel layer. The gel layer was formed by mixing dry polymers and water. Such a process resulted in the change of mixing entropy (thermodynamics). Flory–Huggins theory is a mathematical model of the thermodynamics of polymer solutions which takes account of the great dissimilarity in molecular sizes in adapting the usual expression for the entropy of mixing^32^. Thermodynamics of a gel layer or a polymer solution can be generally discribed by Flory-Huggins theory, where the volume of a polymer system is defined as a lattice which is divided into microscopic subspaces (called sites) of the same volume^29^,^30^. Figure 1b shows the schematic of the cross-section of a gel layer. According to this theory, in the case of a polymer solution, the solvent (water) molecules are assumed to occupy single sites, while a polymer chain of a given type, i, occupies sites n_i_. The repulsive forces in the system are modelled by requiring each lattice site to be occupied by only a single segment (Fig. 1b). Assuming random and ideal mixing, the combinatorial entropy of mixing (Δ*S*_*m*_) to form a gel layer can be given


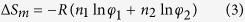


where R is the universal gas constant; n_1_ and n_2_ are the molar number of water and polymer, respectively; *φ*_1_ and *φ*_2_ are the volume fraction of water and polymer, respectively; the subscripts “1” and “2” denote water and polymer, respectively.

According to Fig. 1b, the enthalpy change (Δ*H*_*m*_) is equal to the energy change per polymer monomer-solvent interaction multiplied by the number of such interactions as expressed by


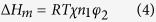


where T is absolute temperature, *χ* is the Flory-Huggins interaction parameter.


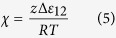


where z is the coordination number; Δ*ε*_12_ is the net energy associated with two neighboring lattice sites of the different polymer segments for the same type or for the different types of polymer chains. The Flory-Huggins interaction parameter gives a measure of the interaction of the polymer chains with the solvent molecules. By definition, the quantity *kTχ* (k is the Boltzmann constant) is the average change in energy when a solvent molecule is transferred from pure solvent to pure, amorphous polymer^29^. For many systems, *χ* has been found to increase with polymer concentration and decrease with temperature with a dependence that is approximately linear with, but in general not proportional to, 1/T. Whereas, *χ* is generally insensitive to the molecular weight of the polymer, except for systems with low molecular weight polymers^33^. *χ* value can be commonly determined by measurements on dilute solutions, especially osmotic pressure measurements. Meanwhile, data of this parameter for lots of polymer-water systems can refer to literature^33^.

Combining with Eqs 3 and 4, Gibbs free energy change (Δ*G*_*m*_) accompanying mixing at constant temperature and pressure can be given by


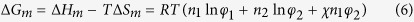


Considering *φ*_1_ + *φ*_2_ = 1, the chemical potential change (Δ*μ*_*m*_) of mixing can be obtained by


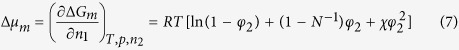


where N is the degree of polymerization. For a cross-linked polymer, N = ∞.

It is implicit that chemical potential change during gel layer formation process can be discribed by Flory-Huggins theory. The feasiblity of this theory has been well verified in literature^32^,^34^,^35^. Wheareas, nobody used this theory to discribe filtration process up to date. Actually, from chemical potential viewpoint, filtration through a gel layer can be regarded as an inverse process of gel formation. As shown in Fig. 1a, there are two chemical potential gradients during the process of water passing through a gel layer. The first gradient between supernatant and gel top layer is likely a spontaneous process as chemical potential of water in supernatant is relatively higher than that in gel layer. Herein, we focused on the second gradient between gel bottom layer and permeate. Although other filtration resistances such as membrane resistance and Carman-Kozeny induced resistance will contribute to the total resistance when a gel on membrane surface was subjected to filtration, these filtration resistances can be ignored as compared with the high total gel filtration resistance. For simplification, only the resistance induced by chemical potential gradient was considered for modeling. It can be seen from Fig. 1a, the chemical potential of water in gel layer is rather lower than that of permeate. Obviously, dragging water from low chemical potential side to high chemical potential side should provide extra energy to overcome the chemical potential gradient.

While it is apparent that there exists a chemical potential gradient between gel layer and permeate of MBR, how to link this chemical potential gradient to filtration parameters is still a challenge, and has not been investigated previously. Based on thermodynamics principle, the chemical potential gradient can be opposed by increasing applied filtration pressure (ΔP)^23^. At a certain point, the equality of chemical potentials between gel layer matrix and MBR permeate is reached. In this study, the following equation was considered to link chemical potential change to the applied pressure.


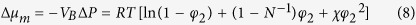


where V_B_ is the molar volume of solvent, which approximates to molar volume of the solution (V) for a dilute solution. Combining with Darcy’s law (Equation1), Eq. 8 can be transformed as:


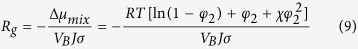


The SFR of gel layer can be calculated by including the filtration resistance and accumulated mass (m_g_):


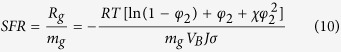


It can be seen from Eqs 9 and 10 that the proposed model and mechanism is strongly pertinent to filtration resistance of gel layer, a parameter representing membrane fouling. Although this study started from the idea that that water has a low chemical potential in the gel layer than in the permeate solution, proposing and verifying the entire mechanism for high resistance of gel layer involved extensive efforts as shown in Eqs 1, 2, 3, 4, 5, 6, 7, 8, 9, 10 and verification section. It should be noted that Eqs 9 and 10 are independent of operational modes including constant flux and constant pressure mode. For a given gel layer, water passing through it needs overcome extra resistance induced by chemical potential variation. On a wide scale, once a pressure is given, the flux is also constant according to Darcy’s law because filtration resistance is relatively constant. In this study, it was deduced that certain part of SFR of gel filtration was resulted from chemical potential gradient between gel bottom layer and permeate. Thus, the factors affecting this chemical potential gradient will affect SFR of gel layer. Obviously, these factors include gel properties such as gel concentration and Flory-Huggins interaction parameter. Equations 9 and 10 provide approaches to estimate the filtration resistance and SFR induced from Flory-Huggins based mechanism during filtration through a gel layer, respectively. Previous study also proposed an osmotic pressure effect during cake layer filtration^36^. The osmotic pressure was originated from the concentration polarization under the special conditions of channels in the cake layer. It can be seen that the mechanism proposed in this study is totally different from that study. The findings in this study are significant as linkage of filtration parameters to gel thermodynamics is first implicitly revealed.

### Simulation of Flory-Huggins model

The SFR induced from Flory-Huggins based mechanism during filtration through a gel layer can be simulated if the related parameters can be determined based on Eq. 10. Considering the complicated components of real gel layer, agar was used as model foulant for gel layer formation. 50 ml agar gel solution (0.03 g·L^−1^) was subjected to filtration to form a gel layer in this study. Simulation was performed on the agar gel layer. The parameter values in this situation as listed in Table 1 were used for simulation. J value was experimentally determined. *χ* value of 0.40 was set for agar-water system according to Colfen^37^. In this study, *φ*_2_ value was determined to be about 0.10. While some thermodynamic properties of the agar gel layer were involved in this study, these thermodynamic properties were just used to simulate filtration resistance of gel layer based on the proposed model.

By using the parameter data listed in Table 1, the SFR induced from Flory-Huggins based mechanism was simulated to be 5.31 × 10^18 ^m·kg^−1^. This value was very high for foulant layer filtration. Obviously, such a mechanism can theoretically explain why gel layer generally possesses unusually high SFR. This study definitely focused on revealing the mechanism underlying high filtration resistance of gel layer rather than other fouling phenomena, and the proposed mechanism is only applicable to predict fouling behavior of filtration resistance of gel layer. Some well-known behaviors of membrane fouling (e.g. limiting flux and dependence on foulant concentration and pressure) under certain conditions can be specifically explained by the models proposed by the literature studies^38^,^39^,^40^.

**Figure 2:**
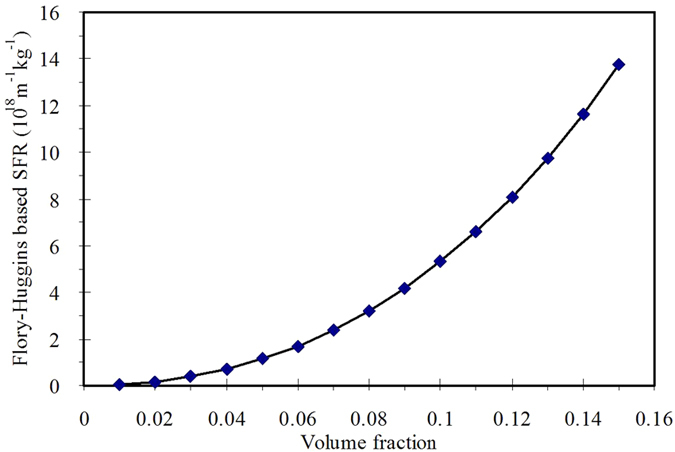
Variation of the Flory-Huggins based SFR of gel layer with volume fraction of agar in gel (Flory-Huggins interaction parameter *χ* = 0.40, agar dry weight m_g_ = 1.5 × 10^−6 ^kg).

Figure 2 shows the dependence of SFR induced from Flory-Huggins based mechanism on volume fraction of the agar gel. It can be seen that the induced SFR increases exponentially with volume fraction of polymer in gel layer, indicating that volume fraction of polymer is a key parameter in the Flory-Huggins based mechanism. In the range of volume fraction of 0.02–0.15, the induced SFR ranges at level of 18–19 orders of magnitude. The simulation suggested that the SFR caused by Flory-Huggins based mechanism could be extremely high, and should be a major contributor to the total SFR of gel layer during filtration.

**Figure 3:**
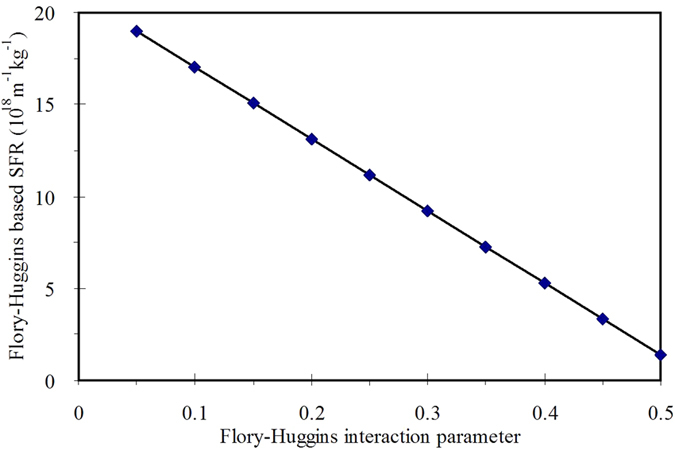
Variation of the Flory-Huggins based SFR of gel layer with Flory-Huggins interaction parameter (volume fraction *φ*_2_ = 0.10, agar dry weight m_g_ = 1.5 × 10^−6^ kg).

Figure 3 shows the effect of Flory-Huggins interaction parameter on the SFR induced from Flory-Huggins based mechanism. The induced SFR decreases linearly with the increase in Flory-Huggins interaction parameter. When Flory-Huggins interaction parameter ranges within 0.05–0.5, the induced SFR also ranges at level of 18–19 orders of magnitude.

It can be also seen from Eq. 10 that pH and ionic strength of the solution almost have no effect on SFR of agar gel layer because these two parameters are not included in the equation. This means that SFR of agar gel layer will not significantly change when pH and ionic strength of the solution are changed.

### Verification of Flory-Huggins based mechanism and model

This study revealed a novel membrane fouling mechanism regarding gel layer filtration. Such a mechanism was suggested to result from the chemical potential gradient between gel layer matrix and water permeate. The chemical potential gradient was considered to be equal to the chemical potential change accompanying mixing of dry polymer and pure water. If the mixing (gel formation/swelling) process can be described by Flory-Huggins theory, as an inverse process, the filtration process of gel can be also described by Flory-Huggins theory. The feasiblity of this theory in gel formation/swelling process has been well verified in literature^32^,^34^,^35^. In this regard, it is no doubt that such a mechanism exists in gel filtration process as gel filtration process can be considered as a kind of inverse process of gel formation.

Although this study mainly focused on deduction of the mathematical model and the new mechanism regarding gel layer filtration, verification of this mechanism was also conducted. According to the model deduced in this study, the filtration resistance induced by this mechanism should be the major contributor to the total filtration resistance of the gel layer. Another deduction from this mechanism is that this mechanism is highly affected by volume fraction of polymer and Flory-Huggins interaction parameter, but is not related to some other parameters such as pH and ionic strength. If these deductions are verified, the hypothesised mechanism should be correct, and the deduced model is feasible.

The mechanism was verified by conducting series of batch filtration tests by using different gel solutions under different conditions. Experimental results showed that the filtration resistance of gel layer linearly increased with gel layer thickness, while SFR of gel layer was irrelevant to gel layer thickness. These results can be explained by the proposed mechanism as gel layer thickness linearly increases Δ*G*_*m*_ accompanying mixing, but doesn’t affect the Δ*μ*_*m*_ of mixing, indicating the real occurrence of this mechanism in gel layer filtration process.

Filtration through agar gel layer under certain conditions in this study showed that the total SFR of gel layer was about 5.68 ± 0.43 × 10^18 ^m·kg^−1^ (4 measurements). In contrast, the SFR induced from Flory-Huggins based mechanism under same conditions was simulated to be 5.31 × 10^18 ^m·kg^−1^ based on the deduced model, indicating that most SFR of agar gel layer was originated from Flory-Huggins based mechanism. Table 2 compares the SFR of gel layer under different pH and ionic strength conditions. One-way ANOVA analysis confirmed that there was no significant difference in SFR between different values of pH (p > 0.05 for all the tested conditions) and ionic strength (p = 0.244). This result indicated that pH and ionic strength had no significant effect on the SFR of gel. Therefore, the two deductions drawn directly from Flory-Huggins based mechanism were satisfactorily verified by batch filtration tests, highly demonstrating the correctness and feasibility of the proposed novel mechanism and model for gel layer.

**Figure 4:**
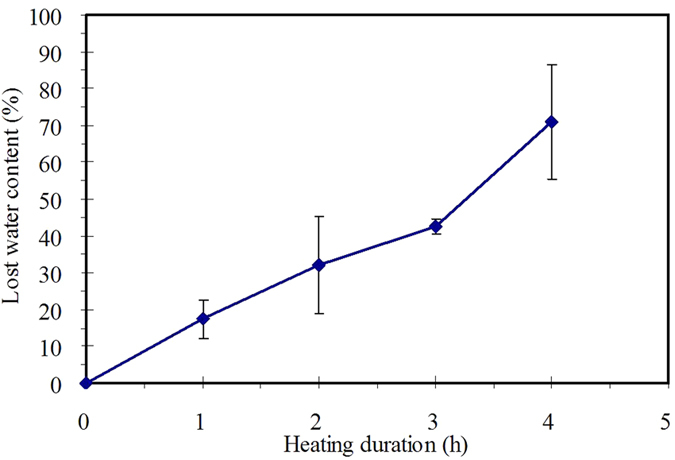
Variation of lost water content of an agar gel with heating duration (agar gel concentration = 3 g·L^−1^, oven temperature = 35 °C).

Verification of this mechanism was also performed by other experiments. The chemical potential change can be also illustrated by heating process of gel. In heating process, as compared with water vapour, the chemical potential of water in gel matrix is rather low. Dragging water from gel matrix to water vapour side also need overcome a chemical potential gradient. Figure 4 shows the dependence of the lost water content in agar gel on the heating duration. The lost water content in agar gel increased linearly with heating duration, indicating that heating can provide continuous force to opposite the chemical potential gradient. As essential of Flory-Huggins based mechanism is the chemical potential change accompanying filtration, such a experiment can serve as an instance verifying the real occurrence of this mechanism.

**Figure 5:**
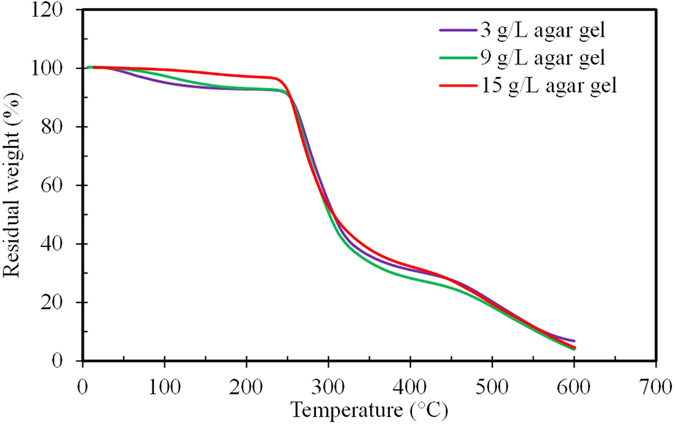
Variation of residual weight of gels with temperature in TGA analysis.

Figure 5 shows variation of residual weight of gels with temperature in TGA analysis. All samples show a weight loss (4.9% for 3 g/L gel, 2.7% for 9 g/L gel, and 0.5% for 15 g/L gel) between 0 and about 100 °C due to the free water loss. Because the agar gels were dried in oven for 12 hours before analysis, most of the free water has been evaporated, that is the reason why just a little weight loss observed during 0 to 100 °C. After about 230 °C, all the samples show a much accentuated mass loss. The results clearly showed that water in the gel has different energy. The water in gel can be classified into free water and bound water. It was reported that bound water to polymeric chains like extracellular polymeric substances (EPSs) was higher than 10 g bound water/dry weight (DW)^41^. Dragging bound water from gel layer need provide extra energy to overcome the energy difference between free water and bound water. At same time, for gel system, carrying bound water in polymer matrix also needs an extra energy. It is argued that the mixing chemical potential variation provides such a force to bind water surrounding the surface of polymers because in this way the chemical potential of the gel system is lowest. According to the law of energy conservation, the experiments can serve as direct evidence for this mechanism.

It is worth noting that, a general operation in mechanism study is adopting model substances to simulate real gel although agar gel layer is not the real gel layer formed in the MBR. By this means, we can focus on the main subject and exclude other potential effects. While there are differences between real gel and model gel, the general structure of gel layers, and the change trend of their properties with affecting factors should be similar (e.g., their surfaces all mainly consist of biopolymers, and their surface properties all are affected by some factors). This study focuses on the later aspects. The basic laws (e.g. chemical potential change trend) revealed in this study are general, which are regardless of real gel or model gel, because the derivation process of the conclusions in this study is based on the general characteristics of gel layer. Therefore, the results obtained in this study have general significance.

## Additional Information

**How to cite this article**: Lei, Q. *et al*. A novel insight into membrane fouling mechanism regarding gel layer filtration: Flory-Huggins based filtration mechanism. *Sci. Rep.* **6**, 33343; doi: 10.1038/srep33343 (2016).

## Figures and Tables

**Table 1:** Parameter values in the gel layer filtration process for simulation.

Parameter	Value for simulation	Unit
R	8.314	kg·m^2^·s^−2^·mol^−1^·K^−1^
T	298	K
V_B_	1.8 × 10^−5^	m^3^·mol^−1^
σ	1.0 × 10^−3^	kg·m^−1^·s^−1^
J	2.35 × 10^−5^	m^3^·m^−2^·s^−1^
*φ*_2_	0.10	—
*χ*	0.40	—
m_g_	1.5 × 10^−6^	kg

**Table 2:** SFR of gel layer under different pH and ionic strength of solution.

conditions	number of measurements	SFR of gel (10^18 ^m·kg^−1^)	significance difference^a^
condition 1# pH = 3.0	4	5.46 ± 0.49	No (Sig_12_ = 0.476)No (Sig_13_ = 0.663)No (Sig_14_ = 0.792)No (Sig_23_ = 0.826)No (Sig_24_ = 0.358)No (Sig_34_ = 0.635)
condition 2# pH = 5.0	4	5.41 ± 0.76	
condition 3# pH = 7.0	4	5.68 ± 0.43	
condition 4# pH = 9.0	4	5.54 ± 0.27	
condition 1# ionic strength = 0 mol·L^−1^	4	5.74 ± 0.31	No (Sig_12_ = 0.244)
condition 2# ionic strength = 0.035 mol·L^−1^	4	5.49 ± 0.22	

^a^Sig. value shown in parentheses, and subscript “mn” means comparing condition m# and condition n#.
